# Tension wood structure and morphology conducive for better enzymatic digestion

**DOI:** 10.1186/s13068-018-1043-x

**Published:** 2018-02-16

**Authors:** Daisuke Sawada, Udaya C. Kalluri, Hugh O’Neill, Volker Urban, Paul Langan, Brian Davison, Sai Venkatesh Pingali

**Affiliations:** 1 Department of Bioproducts and Biosystems, School of Chemical Engineering, Aalto University, Espoo, Finland; 2 Biosciences Division and BioEnergy Science Center, Oak Ridge National Laboratory, 1 Bethel Valley Road, P.O. Box 2008, Oak Ridge, TN 37831 USA; 3 Neutron Scattering Division, Oak Ridge National Laboratory, 1 Bethel Valley Road, P.O. Box 2008, Oak Ridge, TN 37831 USA; 4 Neutron Sciences Directorate, Oak Ridge National Laboratory, 1 Bethel Valley Road, P.O. Box 2008, Oak Ridge, TN 37831 USA

**Keywords:** Tension wood, Poplar, Small-angle neutron scattering, SANS, Wide-angle X-ray diffraction, WAXD, Coalescence of cellulose microfibrils

## Abstract

**Background:**

Tension wood is a type of reaction wood in response to bending or leaning stem as a corrective growth process. Tension wood is formed by both natural and man-made processes. Most attractively, tension wood contains higher glucan content and undergoes higher enzymatic conversion to fermentable sugars. Here, we have employed structural techniques, small-angle neutron scattering (SANS) and wide-angle X-ray diffraction (WAXD) to elucidate structural and morphological aspects of tension wood conducive to higher sugar yields.

**Results:**

Small-angle neutron scattering data exhibited a tri-modal distribution of the fibril cross-sectional dimension. The smallest size, 22 Å observed in all samples concurred with the WAXD results of the control and opposite side samples. This smallest and the most abundant occurring size was interpreted as the cellulose elementary microfibril diameter. The intermediate size of 45 Å, which is most pronounced in the tension side sample and consistent with WAXD results for tension side sample, indicates association of neighboring elementary microfibrils to form larger crystallite bundles. The largest size 61 Å observed by SANS was however not observed by WAXD and therefore associated to mesopores.

**Conclusions:**

Structure and morphology of tension wood is different from control wood. Cellulose crystallinity increases, lignin content is lower and the appearance of mesopores with 61 Å diameter is observed. Despite the presence of higher crystalline cellulose content in tension side, the lower lignin content and may be combined with the abundance of mesopores, substantially improves enzyme accessibility leading to higher yields in cellulose digestion.

**Electronic supplementary material:**

The online version of this article (10.1186/s13068-018-1043-x) contains supplementary material, which is available to authorized users.

## Background

Plant lignocellulose, the most abundant renewable resource material, is an intricately intertwined complex of three primary biopolymers-cellulose, hemicellulose and lignin. The primary function of the biopolymers is to form the structural framework of plants. These biopolymers mainly reside in the plant cell walls, primary and secondary. The secondary cell wall is further classified as S1, S2, S3 and G-layer [1]. The structure and arrangement of these biopolymers differ by plant species based on the chemical compositions, the fundamental structure of each biopolymer, and their distribution in the different layers. A typical composition of dry plant cell wall is 45% cellulose, 20% hemicellulose and 30% lignin, 5% ash and other contents [2]. The regular arrangement of cellulose strands in the elementary cellulose fibril follows cellulose I_β_ unit cell in both tension and control wood [3]. The crystal structure of cellulose I_β_ including hydrogen positions has been determined using a combination of high-resolution X-ray and neutron fiber diffraction [4]. Cellulose I chains pack in thin long bundles called ‘microfibrils’ or ‘elementary microfibrils’ [5–7]. Elementary microfibrils associate to form “macrofibrils” observed in both the primary [8] and secondary cell walls [9]. The cell walls consist of helically patterned cellulose microfibrils encapsulated by a matrix of hemicellulose and lignin. The cellulose microfibrils grow in a direction that deviates from the longitudinal axis of the cells. This angle called the microfibril angle, MFA, for the dominant layer S2 of the secondary cell wall is typically between 5° and 30°, while for the S1 and S3 layers, it is 50–70° and > 70°, respectively [1, 10].

Tension wood is a type of reaction wood formed on the upper side of bending/leaning steams in woody angiosperms such as *Populus*. This newly produced wood as a reaction to environmental conditions has no apparent impact on health. Tension wood formation in woody angiosperms is characterized by phenotypic changes such as production of more xylem fiber cells, thicker secondary walls, enhanced cellulose biosynthesis, reduced lignin biosynthesis, and reduced recalcitrance [2, 11–13]. Since these cellular level characteristics are also desirable in lignocellulosic biomass employed in cellulosic fuels or bioproduct production, tension wood formation presents an excellent model for fundamental structural, metabolic and molecular studies related to informed design, optimization and improvement of feedstocks. Specifically, tension wood has ~ 5–10% less lignin [2, 14] and up to 10% more cellulose content depending on the strength of the tension stress [15]. Enzymatic digestion of tension wood results in relatively higher sugar yields suggesting optimal conversion may be possible without the need of thermochemical pretreatment. For example, a field study of short rotation coppice willow when grown under environmental tensional stress (i.e., exposed to high winds) had greatly improved glucose release yields (up to fivefold) without any pretreatment as compared to a tensional stress-protected environment (i.e., sheltered from winds) [16]. In a separate study, glucose yield from tension wood of *Populus tremula* × *alba* increased threefold compared to control wood [2]. Consequently, tension wood has the potential to be a highly desirable source to produce biofuels [17]. A greater understanding of the molecular, chemical and structural properties associated with tension wood formation will expand the critical knowledge base needed for development of advanced biomass improvement strategies [2, 18, 19].

Realizing detailed characterization of tension wood is limited by the difficulty of separating scattering contributions from the wood cells in the primary and the secondary walls and the additional G-layer. Besides, it is difficult to extract cellulose fibrils from a specific layer without disrupting its native structure. However, by studying slices of intact plant stems that maintain the plant’s natural growth orientation, we greatly alleviate such constraints. In this study, small-angle neutron scattering (SANS) and wide-angle X-ray diffraction (WAXD) studies were performed on intact samples to elucidate structural and morphological details over a wide range of length scales spanning from 1 to 500 nm. Two types of external stress, bending and leaning were applied to poplar wood to produce tension wood samples. Our studies show three distinct sizes in tension, opposite and control wood corresponding to elementary fibril units, naturally associated cellulose fibril aggregates, and mesopores. Interestingly, the trends in the reactional structural changes in the tension wood cell walls exhibit similarities to the salient changes observed due to acidic (dilute) pretreatment [20].

## Methods

### Material

Greenwood stem cuttings of the *Populus tremula* × *alba,* clone 717, plants were propagated and grown in soil under greenhouse conditions as described previously [2, 12] at Oak Ridge National Laboratory. Following 6 months of growth, three replicates of plants were included in the 3-week tension stress experiment. For bending stress (B), the main stem was bent with shoot tip held at 90° [2], and for leaning stress (L), the pot and the main stem was held at an approx. 45° angle, relative to the original main axis of the stem. The control stems (C) were held erect for the duration of the experiment.

### Sectioning and sample preparation

From the bottom section or base of the plant, 1-cm-length stems were cut and used to prepare samples by sectioning. Using a sliding microtome instrument, samples were tangentially sectioned into slices of 200 μm thick by 5 mm length by 1 mm width. Although all samples were prepared in the same manner, for tension and opposite side samples, first few slices representing layers formed prior to the application of stress were discarded. Sliced samples intended for SANS studies were immediately immersed in 100% D_2_O solvent to avoid dehydration. While the samples intended for X-ray diffraction were dried in 80 °C oven to remove solvent and minimize solvent scattering.

### X-ray diffraction

X-ray diffraction data were collected in the transmission mode setting of a rotating anode X-ray generator, MicroMAX-007HF (RIGAKU) operated at 30 mA and 40 kV, using Cu *K*_*α*_ radiation (*λ* = 1.5418 Å). The sample was fixed in position using a block of clay on a goniometer with the sliced face perpendicular to the X-ray beam. The sample-to-detector distance was fixed at 100 mm. Multiple locations on the same sample were exposed for 300 s each to account for spatial heterogeneity of the sample. Diffraction data showing anisotropic scattering image was converted to 1D equatorial and meridional curves and the crystalline characteristics such as crystal size and crystallinity were estimated after peak fitting as detailed in Additional file 1. Concisely, crystal size was determined by the Scherrer equation and crystallinity was determined from the ratio of crystalline area to the total area under the curve.

### Small-angle neutron scattering

**Fig. 1:**
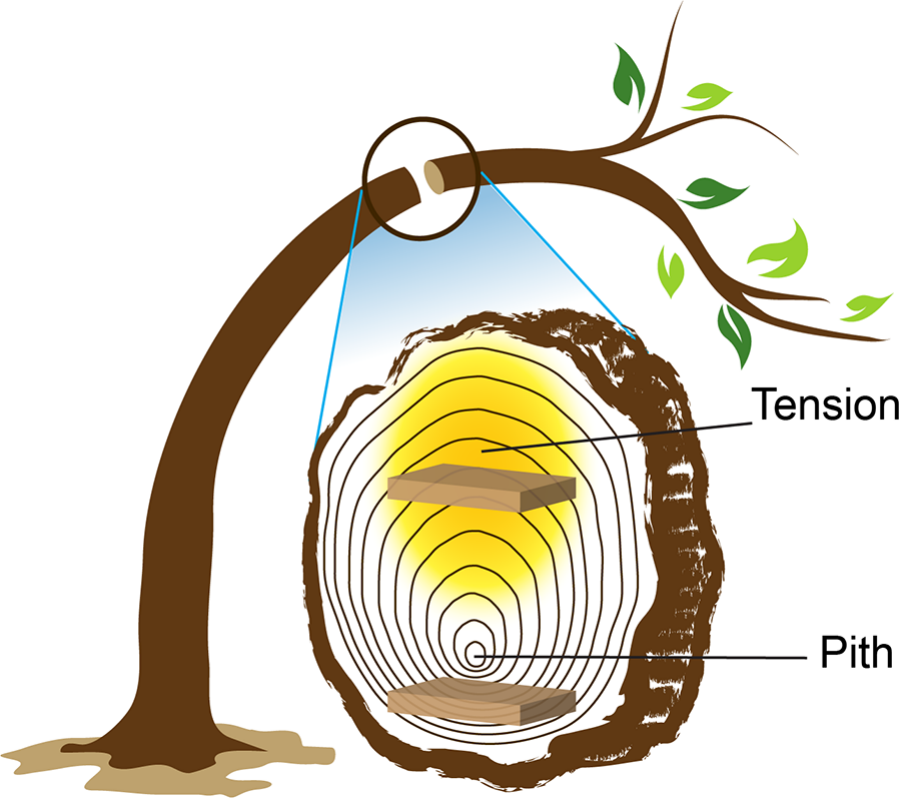
Illustration shows samples for scattering experiments obtained from the *Populus* branch when subjected to bending stress. The two intact slices of tension and opposite wood were prepared from above and below the pith, respectively

Small-angle neutron scattering was carried out at the CG-3 Bio-SANS instrument [21] located in the High Flux Isotope Reactor (HFIR) in Oak Ridge National Laboratory (ORNL). The samples soaked in 100% D_2_O solvent were aligned in the meridional direction in 0.5-mm-thick quartz cells with detachable cell walls (Hellma model #106-QS 0.5 mm); i.e., the wood cell longitudinal direction was aligned upright, Fig. 1. Three different instrument configurations were used to span a wave-vector range, *Q* of 0.003–0.7 Å^−1^, achieved by utilizing sample-to-detector distances of approx. 1.1, 6.8 and 15.3 m at 6 Å neutron wavelength. Wave vector is expressed as *Q* = (4*π*/*λ*) sin*θ*, where 2*θ* is the scattering angle and *λ* is wavelength. The instrument resolution was defined by circular aperture diameters of 40 mm as source and 12 mm as sample aperture separated by an approximate distance of 17.4 m. An area detector, 1 m × 1 m GE-Reuter Stokes Tube detector, was offset horizontally by 0.35 m perpendicular to the beam direction to maximize the *Q*-range accessed for each instrument configuration.

### SANS data reduction and analysis

The anisotropic structural shape of fibrils in plant cells generated an anisotropic 2D scattering pattern for most samples. The raw 2D images were processed by normalizing to incident beam monitor counts, correcting for detector dark current, pixel sensitivity and subtracting scattering contribution from quartz cell. The angular range of the fibril orientation was determined in the intermediate configuration, sample-to-detector distance of 6 m for each sample, and the same angular range indicative of the fibril orientation was applied for the other two configurations. Two scattering intensity profiles *I*(*Q*) versus *Q* were obtained from the equatorial and meridional sectors of the 2D image (Fig. 1). The scattering intensity in the equatorial sector is dominated by cellulose fibrils along the long dimension of the cell wall (plant’s growing direction). While the meridional sector includes contributions from the cellulose fibrils along the short dimension of the cell wall and matrix co-polymers. The angular distribution in the fibril orientation for tension side of each sample was narrower compared to that obtained for its opposite side and this trend was clearly pronounced in the WAXD data (Additional file 1: Table S1). Even though solvent scattering was isotropic, solvent profiles used for subtraction were re-generated with corresponding fibril orientational angular range for consistency.

Small-angle neutron scattering data was analyzed using the maximum entropy size distribution approach implemented in the IRENA package [22]. The maximum entropy method implemented in the IRENA package is by Skiling and Bryan [23]. This approach was employed to deconvolute size information in the *Q*-range 0.01–0.35 Å^−1^. Small-angle scattering intensity is expressed as [22, 24, 25]

$$I \left( Q \right) = \left| {\Delta \rho } \right|^{2} \mathop \sum \nolimits \left| {F\left( {Q, r} \right)} \right|^{2} V\left( r \right)NP\left( r \right){\text{d}}r$$

where $\left| {\Delta \rho } \right|^{2}$ is the scattering contrast, *F*(*Q*,*r*) is the scattering particle form factor, *V*(*r*) is the particle volume, *N* is the number of particles, *P*(*r*) is the particle radius distribution and *r* is particle radius.

Cellulose forms long fibrils, and therefore, particle shape was approximated to long cylinders with diameters in the range 5–500 Å and length was fixed arbitrarily at 5 μm. The fit, logarithmically binned into 300 intervals, produced a smooth size distribution profile of the particle radius.

## Results

Tension wood was studied to elucidate nano- to submicron structural variations between the tension and opposite sides of the plant and further compared with a control plant (grown under no tension stress).

**Fig. 2:**
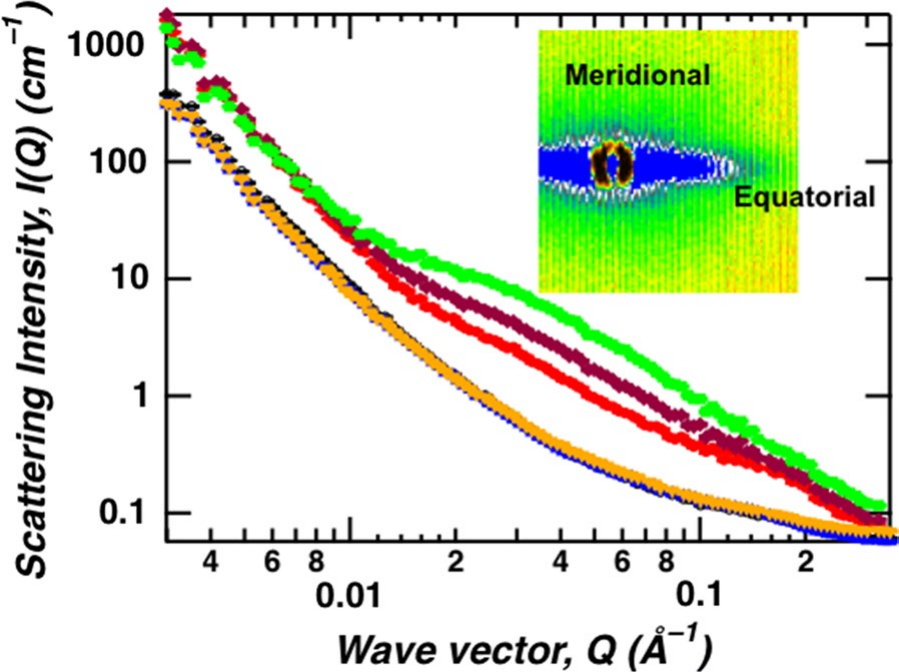
SANS profiles of poplar wood plotted as scattering intensity, *I*(*Q*) vs. wave vector, *Q* for meridional and equatorial sectors. Tension side (green), opposite side (red) and control (purple) show distinct equatorial sector scattering signal compared to the meridional sector scattering signal of the tension side (orange), opposite side (blue) and control (black) samples

The inherent anisotropic shape of cellulose fibrils when arranged in an aligned manner within the plant cell wall produces anisotropic 2D patterns as observed by SANS, especially when intact samples are studied (Fig. 1). The 2D pattern indicates that the cellulose fibrils are vertically aligned and the scattering in the equatorial direction represents the structural features of the cross-sectional dimension such as cross-sectional size and/or distance between neighboring fibrils. Figure 2 shows azimuthally averaged intensity profiles for equatorial and meridional sectors as a function of *Q* for all the samples. The scattering profiles of the equatorial sector for tension side (green squares), opposite side (red dots) and control (purple diamonds) samples differ significantly indicating changes to structure due to the application of tension stress. Based on the sizes involved, these changes are associated with the cellulose fibril structure. Furthermore, our studies suggest that the method of applying tension stress, bending or leaning, cause no measureable difference to the cellulose fibril structure (see Additional file 1: Figure S1). Interestingly, higher variability in the scattering profiles were observed for bending replicates than for leaning replicates which is attributed to variable expression of the tension wood phenotype. On the other hand, the profiles of the meridional sectors for the different samples look the same. The contribution to the meridional scattering is mainly from disordered components such as lignin and hemicellulose and cellulose fibrils aligned horizontally and perpendicular to the beam like ray cells and the shorter side of the cells. Interestingly, tension stress does not cause a measurable change to the structure and arrangement of these components.

**Fig. 3:**
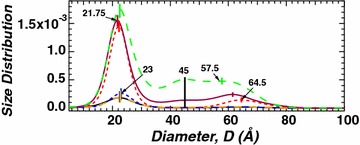
Particle size distribution analysis of equatorial and meridional SANS profiles. The particle shape assumed is a long cylinder. Tension side (green long-dashed line), opposite side (red short-dashed line) and control (purple solid line) show distinct scattering signal on equatorial sector compared to tension side (orange long-dashed line), opposite side (blue short-dashed line) and control (black solid line) samples on meridional sector

Small-angle neutron-scattering data was analyzed by employing particle size distribution using maximum entropy method [22, 24, 25] to consistently extract structural details from all the samples. Particle size distributions extracted from the equatorial sector show significant differences among tension side (green long-dashed line), opposite side (red short-dashed line) and control (purple solid line) samples, while minimal differences were observed for the meridional sector (Fig. 3). For the equatorial sector, the size distribution profiles is a tri-modal distribution with the most intense signal observed for the tension side sample and the least for the opposite side sample. On the other hand, the size distribution for all the samples in the meridional sector is a single peak. This single peak is the same distinct smallest size obtained for the equatorial sector, ~ 22 Å (21.5–23 Å). Although plants exhibit a complex structure with multiple component biopolymers, based on our previous studies [20, 26, 27], the most probable feature responsible for the smallest peak in the size distribution is the cross-sectional dimension of the elementary cellulose fibrils. Hence, WAXD was employed to correlate the cross-sectional crystallite size of the elementary cellulose fibril (or aggregated bundle) with the dimensions observed by SANS.

**Fig. 4:**
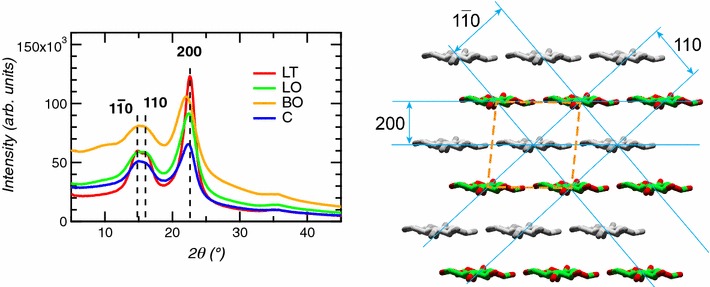
WAXD profiles of tension (leaning, LT), opposite (leaning, LO and bending, BO) and control (C) samples of poplar wood. The three main lattice spacing of the cellulose I_β_ unit cell ($1\bar{1}0$, 110, and 200) indicated in the graph is visually depicted in the schematic (right)

Wide-angle X-ray diffraction intensity profiles represent internal atomic structural dimensions of the cellulose I_β_ unit cell, namely $1\bar{1}0$, 110 and 200 lattice peaks (Fig. 2). The tension side sample shows pronounced lattice peak features and low amorphous background. Pronounced lattice peaks indicate better crystalline order and low background corresponds to a higher crystalline content. The lattice peak 200 for tension side samples (Fig. 4, profile LT) is the sharpest and most pronounced of all samples. Further, the lattice peaks $1\bar{1}0$ and 110 were resolved as individual peaks even though visually the overlap seems significant. Peak fitting [28] results for the degree of crystallinity and crystallite size are summarized in Table 1. All samples were measured in triplicates (Additional file 1: Figure S2).

**Table 1:** Structural parameters extracted from wide-angle X-ray diffraction analysis

	Crystalline area (%)	Crystallite size (Å)			
		$1\bar{1}0$	110	200	Ave.
Bending tension, BT	54 ± 5	31 ± 5	34 ± 4	41 ± 4	35
Bending opposite, BO	28 ± 3	17 ± 1	19 ± 1	30 ± 1	22
Leaning tension, LT	56 ± 3	33 ± 6	33 ± 4	42 ± 5	36
Leaning opposite, LO	40 ± 2	19 ± 1	20 ± 1	32 ± 1	24
Control, C	38 ± 2	20 ± 1	21 ± 2	33 ± 2	25

Crystalline area was estimated from the ratio of total area to the sum of area of the three equatorial peaks ($1\bar{1}0$, 110 and 200)

The results show interesting trends associated with the tension side in comparison to opposite side and control samples of the tension wood. For example, tension side samples form large crystallites with low amorphous content and tension stress causes crystalline nature to propagate further along the $1\bar{1}0$ /110 lattice direction. The ratio between crystallite size of tension side and opposite side for the 200 lattice direction is ~ 1.4 and for $1\bar{1}0$ /110 is higher ~ 1.75. In comparing between opposite and control samples, bending opposite samples show subtle differences to leaning opposite and control samples. The crystalline area of bending opposite sample is 40% lower and similarly, the crystallites in all directions are smaller than leaning opposite and control samples. These variations could be attributed to variable expression of the tension wood phenotype as mentioned earlier.

Briefly, the structure and morphology of tension wood is obtained by combining the results obtained from SANS and WAXD. WAXD measures the crystalline structure of materials. The position and width of the diffraction peaks represents the repeat structure of the crystalline material and the crystallite sizes, respectively. On the other hand, SANS, in general is sensitive to the inhomogeneity in the material which includes crystalline and non-crystalline features [26]. The 22 Å crystallite size obtained by WAXD (see Table 1) represents the cross-sectional dimension of the cellulose microfibril. Consistent with WAXD observation, the first peak in the size distribution profile of the SANS data (see Fig. 2) is also around 22 Å, and therefore, interpreted to represent the cross-sectional dimension of the cellulose microfibril. Similarly, the 45 Å crystallite size, approximately twice the 22 Å crystallite size, is observed by both WAXD (Table 1) and SANS (Fig. 2) and represents the coalesced cellulose microfibrils. The propensity for coalescence of cellulose microfibrils has been reported in previous studies [20, 27] and particularly pronounced when matrix copolymer content is low [2]. Contrary to 22 Å and 45 Å features, the 61 Å peak observed by SANS (Fig. 2) is absent in the WAXD data (Table 1) implying the 61 Å feature represents a non-crystalline component of the material. Here, the non-crystalline feature is the pore structure in the plant material which has been observed and reported [29, 30].

## Discussion

**Fig. 5:**
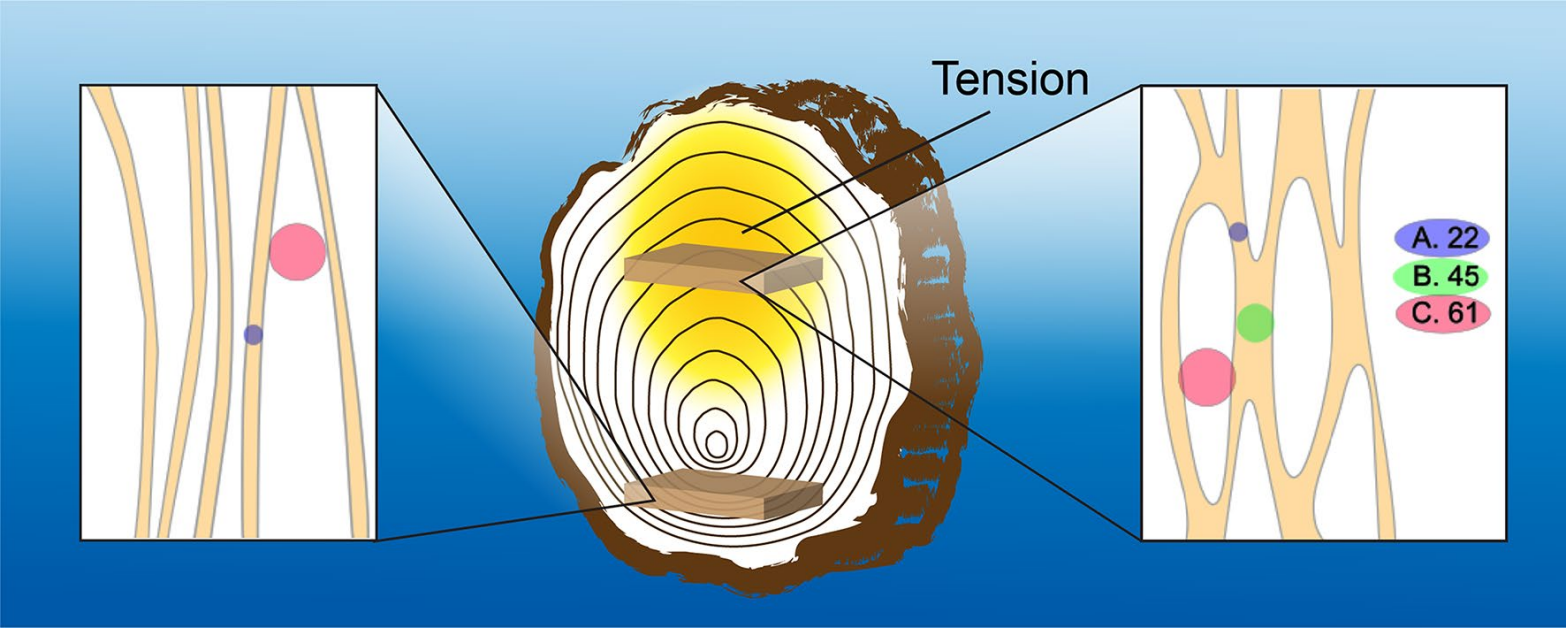
Elementary fibril association model constructed from the size distribution analysis of opposite (bottom of bark), and tension wood (top of bark). Three sizes are associated with cellulose elementary fibrils (A), aggregated fibrils (B) and mesopores (C)

The schematic overview of our findings summarized in Fig. 5 conveys tensional stress promotes formation of cellulose microfibrils with higher crystallinity; coalescence of neighboring cellulose microfibrils; and the presence of mesopores within the cellulose microfibril structure. Consequently, in addition to the diminished composition of lignin in the tension side, the presence of mesopores may also contribute to the higher enzymatic hydrolysis yields.

Small-angle neutron scattering measurements were performed using samples that were minimally perturbed to preserve the natural features of the plant’s internal structure. The orientation of the cellulose fibrils in the different layers, S1, S2, S3 and G of the cell wall are predominantly oriented along the long axis of the stem with a few oriented orthogonally. Consequently, our samples produce cellulose fibril features in both the orthogonal scattering directions but with significantly different intensities. The equatorial scattering is produced from most of the cellulose fibrils that are aligned along the stem’s long axis. While, the cellulose fibril feature in the meridional scattering direction which is significantly diminished is produced by the lower proportion of cellulose fibril aligned orthogonally to the stem’s long axis. Using intact samples allows deconvoluting scattering in the perpendicular directions from the different cell wall layers. The dominant layers, S2 and G, are responsible for the anisotropically enhanced scattering observed in the equatorial direction as compared to layers S1 and S3 that produce weaker scattering in the meridional direction [1]. Analysis of the SANS data of the equatorial sector using the maximum entropy method showed that there are three discrete sizes with the maximum size at approximately three times the size of the smallest diameter, ~ 22 Å. The smallest diameter of 22 Å is similar or slightly smaller than the average size obtained from the three lattice directions for opposite and control woods obtained from WAXD analysis (Table 1). This feature is attributed to the cellulose fibril cross-sectional dimension identified in Fig. 5 as characteristic size A. Unlike the data in the equatorial sector, the scattering profiles of the meridional sector [31] for all the samples are identical suggesting tension does not affect cellulose fibril structure in the S1 and S3 layers. In addition, size distribution analysis results exhibited a single dimension, 22 Å, similar to the smallest diameter of the cellulose fibrils. The similarity in the smallest diameter for both equatorial and meridional scattering profiles indicates that the cellulose elementary fibril structure is identical in all layers of the cell wall.

Tensional stress, in the form of bending or leaning forces has previously been shown to result in aggregation of neighboring cellulose fibrils to form larger fibrils [2], consistent with the findings of this study. Only under tension stress do we observe characteristic size of 45 Å suggesting that tensional stress facilitates coalescence of neighboring fibrils (green dot and labelled B in Fig. 5). We believe the coalescence of adjacent microfibrils mostly occurs in the ‘G-layer’ [32], as G-layer only forms in the tension side of tension wood. The characteristic size of 45 Å is observed by both SANS and WAXD, although SANS results are slightly larger than the results obtained from WAXD.

The equatorial scattering profiles for all samples, control, opposite and tension show the existence of a larger dimension, at 61 Å. Similar to the interpretation of the 45 Å peak, a possible explanation would be the association of multiple elementary fibrils to form a large fibril with an average cross-sectional dimension of ~ 61 Å. However, there is no indication from the WAXD data of a crystallite with this dimension. In addition, the absence of the feature at 45 Å for the opposite side of the same sample renders this scenario to be unlikely because associations of multiple elementary fibrils should include associations of two elementary fibrils needed for 45 Å dimension. A more likely explanation would be scattering contribution from pores in the elementary fibrils (Fig. 5C). It has previously been reported that pores can be found within the cellulose fibril networks of control wood which is consistent with our data that shows this feature in control and tension wood samples. Indeed, mesoporosity has been observed for tension woods of chestnut, lauraceae (*Sextonia rubra*) and poplar with a pore diameter range of 20–500 Å [33] and an average size of 60 Å [34], which is consistent with the data reported here. Further, Grethlein reported the positive correlation between initial hydrolysis rate and the presence of pore volume which has diameter of 51 Å [29]. Tension wood was determined to be more susceptible to enzymatic digestion compared to control wood [2]. This improved digestion was suggested to be associated to the reduced amount of lignin presence, higher S/G ratio and increased accessible specific surface area. And unlike once thought, higher cellulose crystallinity and formation of larger cellulose fibril when observed in isolation could be less significant in hindering enzymatic digestion. Our findings are consistent with these observations. Furthermore, pores conducive for enzymatic access to more cellulose fibrils in tension wood could very well compensate the negative effect of fibril aggregation by increasing enzyme accessibility to the cellulose fibrils.

Interestingly, thermochemical pretreatments cause similar changes to the structure and morphology of the lignocellulosic biopolymers, especially acidic pretreatments. For example, dilute sulfuric acid treatment of switchgrass doubled the cross-sectional diameter of the elementary cellulose fibril, 20–44 Å [26, 27]. Likewise, steam explosion pretreatment displayed strong evidence of fibril aggregation [20]. The current understanding is that acidic pretreatment hydrolyzes hemicellulose and promotes association of lignin biopolymers thereby energetically accelerating the aggregation of cellulose elementary fibrils at reasonably elevated temperatures (> 80 °C) [20, 35]. The naturally occurring structural change due to tensional stress is, therefore, likened to the effect of acidic pretreatment. Evidence of larger cellulose fibrils due to tensional stress has been reported, but the mechanism [36]. Despite the presence of larger fibrils and thinner S2 layers [37] in tension samples, a significantly large proportion of the fibrils are of the smallest diameter. This observation indicates that larger fibrils in tension samples observed by WAXD still maintain the identity of their smallest fibril units, like aggregated crystallites of the smallest cellulose fibril units. Furthermore, the presence of 20% of matrix polysaccharide in G-layer [38] is most probably responsible in preventing the formation of perfect cellulose crystallites [39].

## Conclusions

The effect of tensional stress on the structure and morphology of the biopolymer components in the cell wall of poplar were elucidated by employing SANS and WAXD for *Populus* plant, *Populus tremula* × *alba*. SANS provided multi-scale morphological details ranging from submicron to nanometer scale and WAXD extends further to atomic resolution like packing of cellulose chains within the crystallites. Tension stress, irrespective of bending or leaning results in similar structural features in the tension wood samples. Small-angle neutron scattering indicated the presence of a distribution of sizes with three pronounced peaks. The smallest size 22 Å, interpreted as the elementary cellulose fibril size, was observed in the wood cells of tension, opposite and control woods. However, associations of the elementary cellulose fibrils resulted in the formation of macro-fibril bundles, 45 Å was observed exclusively in the tension wood samples by SANS. The crystalline characteristics of the elementary fibrils and macro-fibril bundles were confirmed with WAXD. The largest size, 61 Å, also just observed in the tension wood sample, is representative of mesopores within the cellulose fibril network. Tension stress promotes elementary cellulose fibril associations resulting in increased crystalline material, an effect similar to thermochemical pretreatments like steam and dilute acid. Consequently, better enzymatic yields for tension wood may also result due to the pronounced presence of mesopores in addition to lower lignin content. Thus, by comparing tension wood structure to thermochemically pretreated wood unlike crystalline nature of cellulose, lower lignin content and may be the abundance of mesopores, play an important role in improving enzymatic hydrolysis of tension wood.

## Additional file

**Additional file 1.** Additional table and figures.
