# Supplementary material for: Tension wood structure and morphology conducive for better enzymatic digestion
Source: Biotechnol Biofuels. 2018 Feb 16;11:44. doi: 10.1186/s13068-018-1043-x (PMC5815229; doi:10.1186/s13068-018-1043-x)
Supplement: Supplementary file 1 — Additional file 1. Additional table and figures. [file 13068_2018_1043_MOESM1_ESM.pdf]

Tension wood structure and morphology conducive for better enzymatic digestion

Daisuke Sawada,[1] Udaya C. Kalluri,[2] Hugh O'Neill,[3] Volker Urban,[3] Paul Langan,[4] Brian Davison,[2] and Sai Venkatesh Pingali[3*]

[1]Department of Bioproducts and Biosystems, School of Chemical Engineering, Aalto University, Finland, [2]Biosciences Division and BioEnergy Science Center, [3]Neutron Scattering Division, [4]Neutron Sciences Directorate, Oak Ridge National Laboratory, 1 Bethel Valley Road P.O. Box 2008, Oak Ridge, TN 37831, USA

[*]Corresponding Author: Sai Venkatesh Pingali; pingalis@ornl.gov; +1-865-241-2424.

Table S1. Orientation parameters from small-angle neutron scattering (SANS) and wide-angle X-ray diffraction (WAXD).

	SANS	WAXD
Bending tension	11.4 ± 2.5	21.5 ± 2.0
Leaning tension	11.4 ± 1.5	21.4 ± 1.7
Bending opposite	14.6 ± 5.7	58.6 ± 14.0
Leaning opposite	17.1 ± 2.0	36.8 ± 6.0
Control	14.0 ± 6.4	29.9 ± 1.2

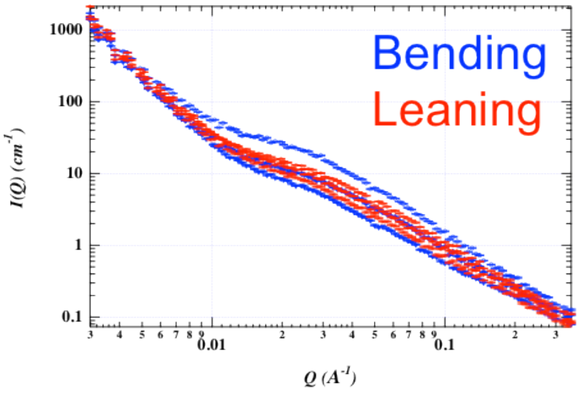

Figure S1. Equatorial SANS intensity profiles from three replicates of bending and leaning samples.

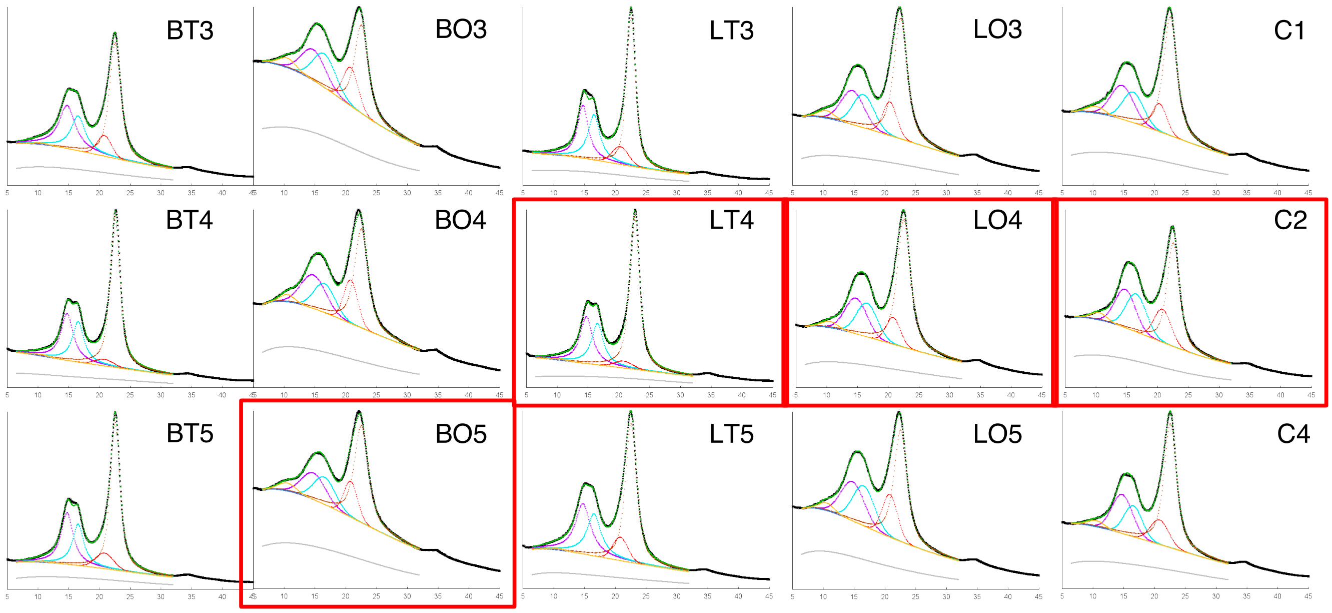

Figure S2. WAXS intensity profiles from three replicates of bending (B series) leaning (L series) and control (C series) samples. The samples indicated with red box are the curves shown in Figure 4. Each profile was fit for background and 4 lattice peaks – $1\bar{1}0$, 110, 012/102, 200.
